# Impact of systemic hypoxia and blood flow restriction on mechanical, cardiorespiratory, and neuromuscular responses to a multiple-set repeated sprint exercise

**DOI:** 10.3389/fphys.2024.1339284

**Published:** 2024-01-31

**Authors:** Robert Solsona, Roméo Dériaz, Simon Albert, Maxime Chamoux, Jaume Lloria-Varella, Fabio Borrani, Anthony M. J. Sanchez

**Affiliations:** ^1^ Institute of Sport Sciences, University of Lausanne, Lausanne, Switzerland; ^2^ University of Perpignan Via Domitia, Laboratoire Interdisciplinaire Performance Santé Environnement de Montagne, Font-Romeu, France; ^3^ University of Rennes, Faculty of Sports Sciences, Rennes, France

**Keywords:** hypoxic training, skeletal muscle, high intensity exercise, vascular occlusion, neuromuscular fatigue, recovery

## Abstract

**Introduction:** Repeated sprint cycling exercises (RSE) performed under systemic normobaric hypoxia (HYP) or with blood flow restriction (BFR) are of growing interest. To the best of our knowledge, there is no stringent consensus on the cardiorespiratory and neuromuscular responses between systemic HYP and BFR during RSE. Thus, this study assessed cardiorespiratory and neuromuscular responses to multiple sets of RSE under HYP or with BFR.

**Methods:** According to a crossover design, fifteen men completed RSE (three sets of five 10-s sprints with 20 s of recovery) in normoxia (NOR), HYP, and with bilaterally-cuffed BFR at 45% of resting arterial occlusive pressure during sets in NOR. Power output, cardiorespiratory and neuromuscular responses were assessed.

**Results:** Average peak and mean powers were lower in BFR (dz = 0.87 and dz = 1.23, respectively) and HYP (dz = 0.65 and dz = 1.21, respectively) compared to NOR (*p* < 0.001). The percentage decrement of power output was greater in BFR (dz = 0.94) and HYP (dz = 0.64) compared to NOR (*p* < 0.001), as well as in BFR compared to NOR (*p* = 0.037, dz = 0.30). The percentage decrease of maximal voluntary contraction of the knee extensors after the session was greater in BFR compared to NOR and HYP (*p* = 0.011, dz = 0.78 and *p* = 0.027, dz = 0.75, respectively). Accumulated ventilation during exercise was higher in HYP and lower in BFR (*p* = 0.002, dz = 0.51, and *p* < 0.001, dz = 0.71, respectively). Peak oxygen consumption was reduced in HYP (*p* < 0.001, dz = 1.47). Heart rate was lower in BFR during exercise and recovery (*p* < 0.001, dz = 0.82 and *p* = 0.012, dz = 0.43, respectively). Finally, aerobic contribution was reduced in HYP compared to NOR (*p* = 0.002, dz = 0.46) and BFR (*p* = 0.005, dz = 0.33).

**Discussion:** Thus, this study indicates that power output during RSE is impaired in HYP and BFR and that BFR amplifies neuromuscular fatigue. In contrast, HYP did not impair neuromuscular function but enhanced the ventilatory response along with reduced oxygen consumption.

## Introduction

In recent years, sprint training methods have gained in popularity in the scientific and sports spheres. Their efficacy at inducing physiological adaptations has been highlighted in several studies (Millet et al., 2019). Importantly, repeated sprint training (RST) enhances repeated sprint ability in athletes (Faiss et al., 2015; Goods et al., 2015; Montero and Lundby, 2017). In combination with hypoxia (HYP), RST is called “RSH” and can boost gains in performance. For example, a study on elite cross-country skiers showed that RSH improved the number of sprints performed during a repeated sprint exercise (RSE) (Faiss et al., 2015). On the other hand, in a study performed on lacrosse players, the percentage increase in peak and mean powers was greater in RSH compared to RST (Kasai et al., 2015). This suggests that the addition of hypoxic stress to RST may improve repeated sprint ability. Interestingly, a sport-specific task to exhaustion was held longer in the tennis players that were assigned to an RSH program (Brechbuhl et al., 2018). Taken together, these results show that RSH can be beneficial for well-trained populations, which is promising since they already have high fitness levels, and their margin of improvement is reduced. Furthermore, adaptations to RSH are driven by cellular mechanisms related to reactive oxygen species, as well as modifications in oxidative and glycolytic metabolism (Kasai et al., 2021). Hematological adaptations can also occur, but results from literature remain inconsistent to date (Camacho-Cardenosa et al., 2022).

The pioneering study by Faiss et al. suggested that the adaptations induced by RSH may be attributed to enhanced muscle perfusion resulting from compensatory vasodilation, especially in fast-twitch muscle fibers (Faiss et al., 2013). Other mechanisms related to oxygen-carrying capacity could be increased capillary density or myoglobin content (Millet et al., 2019). Metabolic adaptations such as faster phosphocreatine resynthesis rates or oxidative enzymes activity could also explain the benefits of RSH. However, the mechanisms behind the beneficial effects of RSH remain underexplored and comparative studies are needed to elucidate the physiological responses between RSH and RST, especially in healthy and moderately trained individuals.

Furthermore, alternative hypoxic methods are emerging in combination with RST protocols. In a study by Mitchell and colleagues, it was found that RST with post-exercise blood flow restriction (BFR) increased $\dot{V}$ O_2_max in trained cyclists (Mitchell et al., 2019). Similar results were found in another study, which also observed an increase in the expression of hypoxia-inducible factor-1α in the muscle biopsies of the BFR group only (Taylor et al., 2016). However, the acute responses need to be compared between RSE under systemic hypoxia and with BFR. It was recently observed that muscle oxygenation is not always reduced during RSE with partial occlusion (Willis et al., 2018). Furthermore, fatigue etiology can differ between BFR and HYP: while the former impairs biceps brachii excitation-contraction coupling after arm RSE, the latter reduces corticospinal excitability (Peyrard et al., 2019). Hence, fatigue accumulation during training represents an additional factor that can impact the subsequent training adaptations. Accordingly, a recent study evaluated the effects of a two-week RST in HYP, NOR and BFR (Giovanna et al., 2022). The authors showed that the group that underwent BFR during recovery periods presented an increase in $\dot{V}$ O_2_peak during a supramaximal test. Thus, conducting RSE with BFR may enhance training adaptations as well, but through slightly different mechanisms compared to HYP. Interestingly, recent research showed that the addition of BFR or HYP to RSE does not influence perceptual responses in active males (Behrendt et al., 2023). In this study, the percentage decrement of power output was higher under BFR and HYP compared to NOR. However, HYP increased blood lactate concentration and BFR reduced muscle activity and oxygenation. Conversely, another study found no difference in the power output with BFR and/or HYP (Wang et al., 2023). Also, BFR increased muscle activity and HYP reduced muscle oxygenation. Therefore, the influence of BFR and HYP during RSE remains a subject of debate, given the conflicting results reported in current research.

While systemic hypoxia enables the concurrent training of large groups in a hypoxic setting, the localized hypoxic environment potentially created by BFR and the metabolite accumulation induced by restricted blood flow would represent an alternative stimulus for the working muscles compared to systemic hypoxia. Some studies showed no difference in power output during RSE in HYP and/or BFR compared to NOR (Ienaga et al., 2022; Wang et al., 2023). Conversely, other studies observed a decrease in mechanical output (Willis et al., 2018; Willis et al., 2019b; Peyrard et al., 2019). This might disfavor training adaptations or, conversely, stem from an increased metabolic stress to promote physiological adaptation. Finally, to the best of our knowledge, there is no strict consensus on the neuromuscular responses between systemic hypoxia and local hypoxia during RSE.

Therefore, the objective of the study was to compare the impact of systemic hypoxia (HYP) and local hypoxia (BFR) on mechanical, cardiorespiratory, and neuromuscular responses in moderately trained men during an RSE. The hypotheses were that HYP and BFR would induce lower peak and mean power than NOR. HYP would induce greater cardiorespiratory demands (*e.g.*, ventilation) than the other conditions, BFR would induce higher neuromuscular fatigue. One of the interests of this investigation is to better understand the mechanical and physiological responses to RSE with hypoxia or BFR, especially because these protocols have seen its popularity increase in the last two decades.

## Materials and methods

### Participants

Fifteen healthy young men volunteered to participate in the study (age 25 ± 3 years, height 1.79 ± 0.05 m, weight 72.7 ± 1.3 kg, body fat percentage 12.0% ± 1.8%). They were physically active (i.e., running or cycling, 8 ± 3 h per week), did not perform RSE in their regular training, and were not acclimatized to altitude (i.e., no prolonged sojourn in the last 6 months). The participants were asked not take alcohol, dietary supplements, medication, or ergogenic aids during all the studied periods and 1 month before to avoid interactions. On the day preceding each experiment, a standardized diet (55% carbohydrate, 15% protein, and 30% fat) was proposed to participants. The study procedures complied with the Declaration of Helsinki on human experimentation and were approved by the local ethics committee (CER-VD n°2022-00238). Athletes were fully informed about data collection and gave written informed consent to participate in the study. Inclusion criteria were to be aged 18 to 30, without any chronic illness and to pass the Physical Activity Readiness questionnaire.

### Study design

**FIGURE 1:**
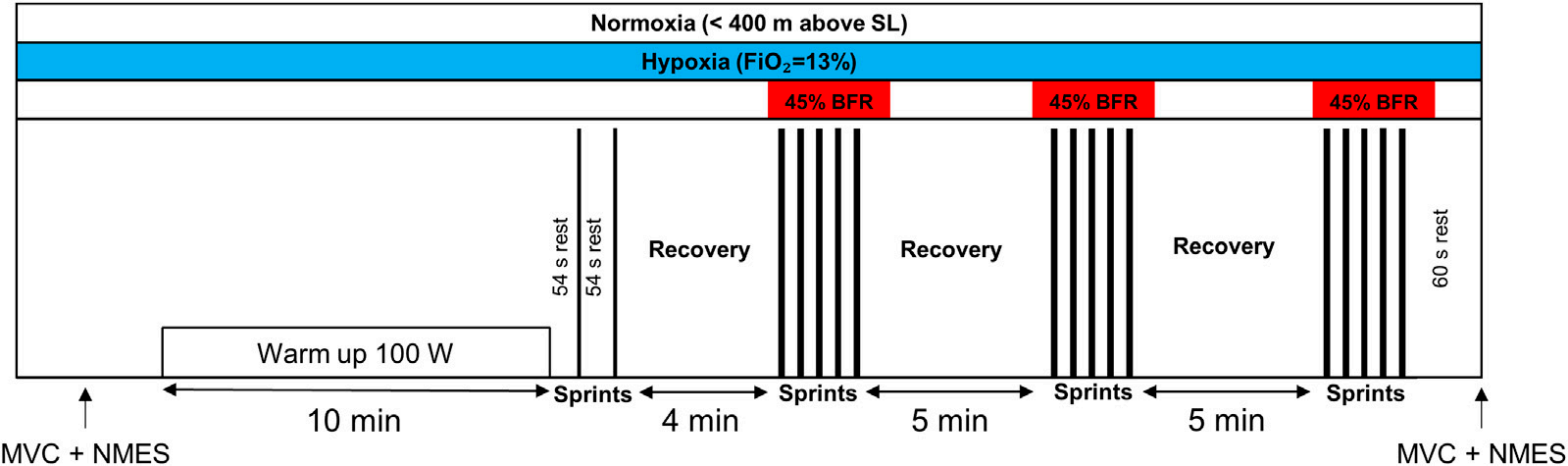
Schematic representation of the exercise protocol. The training session consisted of a 10-min warm-up at 100 W, followed by short recovery periods (i.e., 54 s) preceding two sprints of 6 seconds. After 4 min of passive recovery, participants performed three sets of five 10-s sprints with 20 s of passive recovery. The three conditions are represented at the moment they were applied. The black rectangles represent the sprints. BFR, bilaterally-cuffed blood flow restriction; FiO_2_, fraction of inspired oxygen; MVC, maximal voluntary contraction; NMES, neuromuscular electrical stimulation; SL, sea level.

The study design was a randomized crossover represented in Figure 1. Participants were asked to perform an RSE on three occasions in a random order: NOR (below 400 m above sea level), HYP (simulated altitude with a fraction of inspired oxygen of 13%, ∼4,000 m), and BFR (bilaterally-cuffed blood flow restriction at 45% of resting femoral arterial occlusive pressure during sets) in NOR. To avoid fatigue-related bias, sessions were performed at least 72 h apart. During the rest period between the sessions, participants were instructed not to perform exhausting efforts 48 h before the trials. Normobaric hypoxia was produced by a hypoxic chamber (ATS altitude training, Australia). Systemic hypoxia was applied before the warm-up and was maintained until the electrostimulation test after exercise. Before and 1 minute after RSE, neuromuscular fatigue was assessed with an isometric chair and electrical nerve stimulation during a maximal voluntary contraction (MVC) of the knee extensor muscles. Strong verbal encouragement was given during RSE and during the MVCs to ensure maximal effort. Body composition was assessed during the first visit using bioelectrical impedance analysis (InBody 770, Cheonan, Chungcheongnam, South Korea).

### RSE protocol

Participants completed an RSE protocol on a cycling ergometer (Lode Excalibur Sport, Lode Medical Technology, Netherlands) at a constant room temperature of 24°C ± 1°C and a relative humidity of ∼53%. The session consisted of a 10-min warm-up at 100 W, followed by short recovery periods (i.e., 54 s) preceding two warm-up sprints of 6 seconds and 4 min of rest. Then, three sets of five 10-s sprints with 20 s of passive recovery were completed. The ergometer was used in Wingate mode with a torque of 0.8 N/kg of body weight. Five-minute recoveries were allowed between the sets. Participants were asked to maintain saddle contact to ensure biomechanical reproducibility. Before each sprint, a three-second countdown was indicated by the investigators before a start from a sitting position on the stationary bike. The ergometer was set to stop its rotation in less than 20 s before each sprint to ensure the absence of inertia on the ergometer’s wheel. Of note, participants remained on the cycle ergometer during the rest and recovery periods between the sets.

### Determination of femoral arterial occlusive pressure

The determination of femoral arterial occlusive pressure was performed on the day the BFR trial was conducted. The measurement was performed at least 10 min before the beginning of RSE. Participants were asked to sit on a chair with their right leg at 90° and the cuff (SC12D Rapid Version Cuff, D.E. Hokanson Inc., United States) around the most proximal part of the limb. Cuff pressure was set at 90 mmHg with a rapid inflation system (AG101 Cuff Inflator Air Source, D.E. Hokanson Inc., United States) and progressively increased until complete ischemia was detected with Doppler ultrasounds (EchoWave II 3.4.4., Telemed Medical Systems, Italy). The trial was repeated at least two times for reproducibility with 1 minute of rest. Cuff size was 13 × 85 cm and the same system was used during the trial. The 45% of the highest value obtained was used for the session (83.4 ± 6.5 mmHg): cuffs were inflated 5 seconds before each set and were deflated 20 s after the last sprint of each set. According to preliminary testing, this was the highest pressure that could be tolerated during the present protocol. BFR was applied during the sets only because (i) pre-experimentations showed that continuous BFR was painful and impeded the completion of the protocol, and (ii) to ensure proper recovery before the beginning of the sets.

### Performance variables measurement

Performance variables were assessed during RSE with the software of the cycling ergometer: peak power, mean power, time to reach peak power, fatigue index and percentage decrement score. Of note, the average peak power presented throughout the manuscript corresponds to the average of peak powers from each set. Fatigue index was calculated as follows:

$$Fatigue\;index=\frac{Peak\;power-Minimal\;power}{Peak\;power}\times 100$$

The calculation of the percentage decrement score was made as follows (Girard et al., 2011):

$$Percentage\;decrement\;score=\left(\frac{\left(S1+S2+S3+S4+S5\right)}{S_{best\;}\times number\;of\;sprints}-1\right)\times 100$$

Where S represents the average power of each sprint and $S_{best\;}$ is the most powerful sprint of the set. Hence, fatigue index represents an intra-sprint indicator, whereas percentage decrement score was intra-set.

### Cardiorespiratory responses assessment

Gas exchanges were evaluated during RSE breath-by-breath with a metabolic cart (Quark CPET, COSMED, Italy) that was calibrated before each test. A heart rate monitor (HRM3-SS, Garmin, United Kingdom) was used to determine the heart rate during exercise and rest (HRex/rest), as well as peak heart rate (HRpeak). Peak ventilation ( $\dot{V}$ Epeak), oxygen uptake ( $\dot{V}$ O_2_peak) and exhaled carbon dioxide ( $\dot{V}$ CO_2_peak) were evaluated. Then, accumulated ventilation (ΣVEex/rest), oxygen consumption (ΣVO_2_ex/rest) and exhaled carbon dioxide (ΣVCO_2_ex/rest) were calculated for each set (150 s) and the two inter-set recoveries (280 s each) from interpolated data at 1 Hz, which were filtered with a second order Butterworth filter with a cutoff frequency of 0.1 Hz. This method was previously used for sprint exercises (Solsona et al., 2021). Aerobic energy contribution was calculated from the accumulated oxygen consumption of each set (i.e., 150 s), of which the basal metabolic rate (3.5 mL min^−1^·kg^−1^) was subtracted (Kwan et al., 2004). The accumulated oxygen consumption was then multiplied by the caloric oxygen equivalent (21.131 kJ L^−1^) to obtain aerobic energy consumption (Hebestreit and Beneke, 2007). Aerobic energy contribution was calculated as the ratio of aerobic energy consumption to total work, with an assumed mechanical efficiency of 23% (ÅStrand et al., 1960).

### Neuromuscular fatigue assessment

Before and exactly 1 minute after RSE (i.e., time was standardized with a timer), electrical nerve stimulation during an MVC of the knee extensor muscles was performed. Neuromuscular fatigue was assessed with an isometric chair that allowed the participants to perform MVC with a knee angle of 90°. The torque of the knee extensors was measured with a load cell (STS 2,500 N, sensitivity 2 mV/V and 1.7 mV/N, SJW, China) that was previously calibrated with known masses. The distance between the fulcrum and the point of application (0.54 m) was considered for torque measurement. Participants wore a belt to avoid body displacements during contractions. Electrical nerve stimulation was achieved with a cathode placed at the inguinal triangle (Stimex TENS 32 mm diameter, Stimex, Switzerland) and an anode on the *gluteus maximus* muscle (MyoTrode Premium 50 × 90 mm, Globus, Italy) to stimulate the femoral nerve. The electrodes were connected to a constant current stimulator (DS7AH, Digitimer Ltd., United Kingdom) that produced 1 ms rectangular pulses. Optimal stimulation intensity was determined before each test by progressively increasing the intensity by 20 mA until the produced torque and the amplitude of the M-wave reached a plateau. Then, the value was increased by 120% to ensure the stimulus was maximal. Thereafter, the participants were asked to warm up by performing muscle contractions before having two opportunities for MVC determination (without electrostimulation). At least 1 minute of rest was allowed between trials. The maximal torque value was recorded as a reference value for the superimposed MVC. To guarantee maximum effort during the superimposed MVC before exercise, a difference of less than 5% was allowed with the best previous trial on the same day. If the difference was greater, the participants rested another minute before retrying. Doublets (100 Hz) were induced by a Train/Delay generator (DG2A, Digitimer, United Kingdom) and were sent during the MVC and ∼2 s after the MVC to calculate the activation level (AL) as follows:

$$\text{AL}=100-D*\left(T_{b}/T_{\max}\right)/T_{DTW}*100$$

**FIGURE 2:**
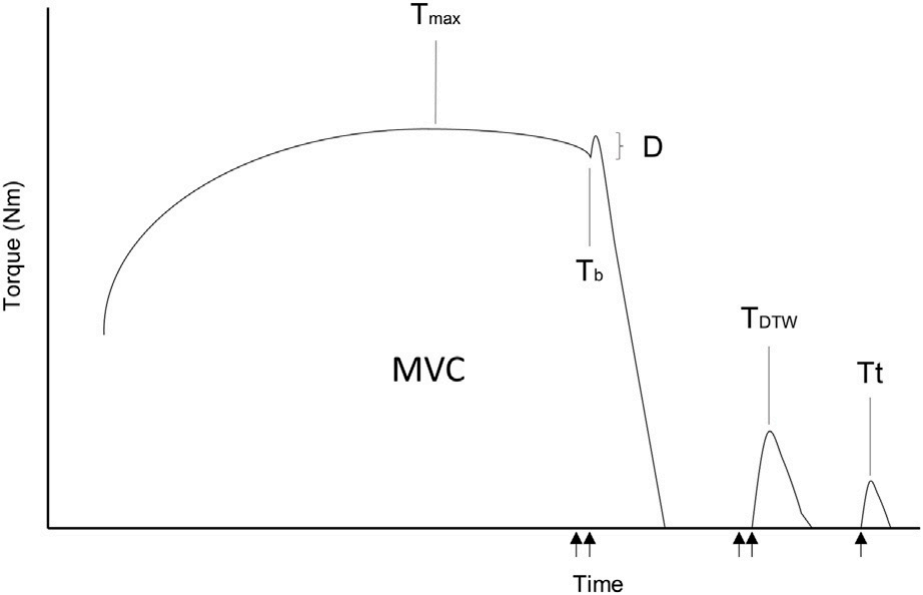
Schematic representation of the electrical nerve stimulation. MVC, maximal voluntary contraction; T_max_, twitch torque during the MVC; T_b_, torque before the superimposed doublet; D, difference between the torque level just before the double twitch and the maximum torque during the double twitch; T_DTW_, torque reached with the isolated doublet; Tt, twitch torque. The arrows represent the electrical stimuli of the femoral nerve (1 ms).

Where D is the difference between the torque level just before the double twitch and the maximum torque during the double twitch, T_b_ is the torque immediately before the doublet, T_max_ is the maximal torque within the MVC and T_DTW_ is the torque reached with the isolated doublet. This formula is used when the stimuli are not delivered exactly at the point when maximal torques are reached (Strojnik and Komi, 1998). An isolated subsequent singlet allowed the calculation of twitch torque (Tt) and the measurement of the maximal M-wave (Mwave_max_) (Figure 2). Electromyography (EMG) electrodes (EMG electrode, 10 mm diameter, Kendall Meditrace 100, Tyco, Canada) were placed at the distal part of the right *vastus medialis* and the reference electrode on the patella. The EMG and torque signals were sampled by an analog-to-digital converter with a frequency of 2 kHz (MP150, Biopac System, United States). Data were acquired and treated with software (AcqKnowledge, Biopac System, United States). After the session, participants were asked to sit on the isometric chair to begin the MVC 1 minute after the last sprint. The percentage difference (ΔMVC, ΔAL, ΔTt and ΔMwave_max_) between pre- and post-values was calculated.

### Statistics

The statistical analyses were performed using Jamovi (Version 1.6.23). After inspecting residual plots, no obvious deviations from homoscedasticity or normality were observed. Therefore, linear mixed models were used with two main effects: condition and time. The fixed effects were the three conditions (i.e., NOR, HYP, BFR), and the three sets (for exercise variables) and the two inter-set recoveries (for recovery variables), as well as their interaction. Of note, the fixed effects were the conditions and time (pre/post RSE session) for neuromuscular variables. Random effects were the intercept and the cluster variable, which corresponded to the participant number. When a main effect was detected, *post hoc* analyses allowed to identify contrasts, and Holms’ correction was used to adjust *p*-values. When the time effect was not possible to evaluate (electrostimulation variables), only the conditions were considered. The significance threshold was set at 0.05. As the effects represent within-subjects comparisons, Cohen’s dz was employed to denote effect sizes (Lakens, 2013): trivial effect d < 0.10, small effect 0.10 ≤ d < 0.50, medium effect 0.50 ≤ d < 0.80 and large effect d ≥ 0.80) (Cohen, 1992; 2016). All data are presented as mean and standard deviation.

## Results

No condition*set interaction was found for any variable (*p* > 0.05).

### Mechanical responses

**FIGURE 3:**
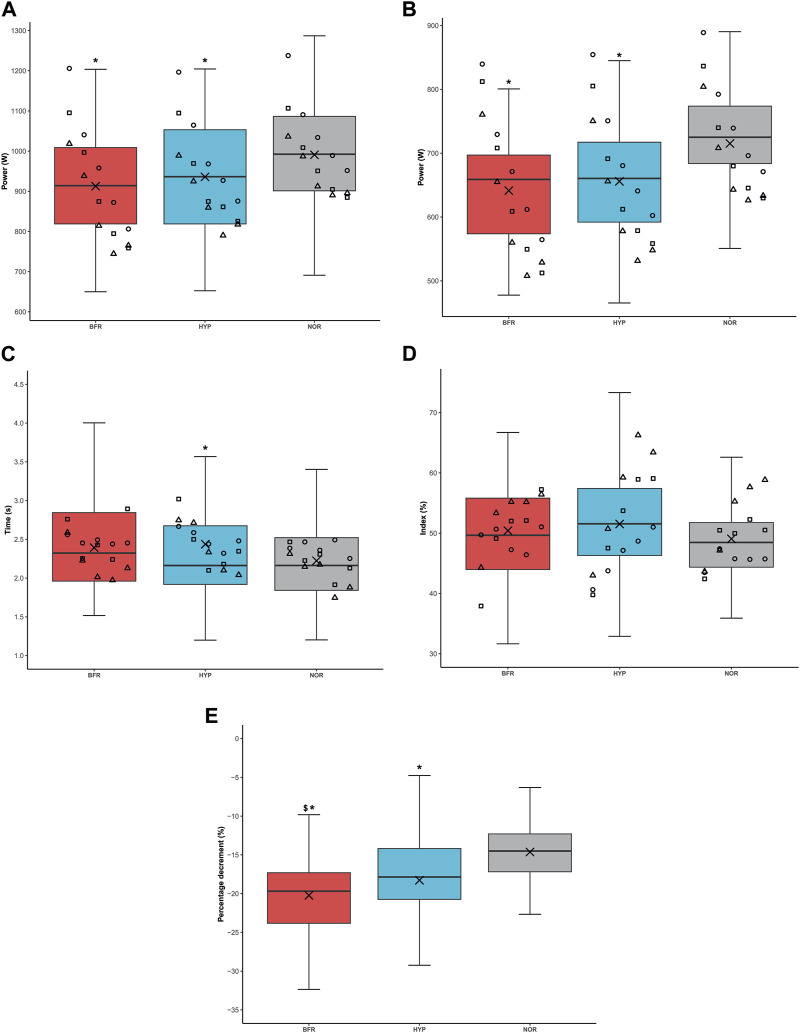
Power output under the different conditions. **(A)** Average peak power output, **(B)** Mean power output, **(C)** Time to reach power output, **(D)** Fatigue index, **(E)** Percentage decrement. BFR: bilaterally cuffed blood flow restriction of the lower limbs at 45% of resting arterial occlusion, HYP: simulated hypoxia (fraction of inspired oxygen = 13%), NOR: normoxia. ^*^: significantly different from NOR (*p* < 0.05), ^$^: significantly different from HYP (*p* < 0.05). Of note, whisker plots represent the quartiles the horizontal line corresponds to the median and the cross is the mean. Circles, squares and triangles represent the sprints of the first, second, and third sets, respectively.

A main effect of condition was found for peak power, mean power, time to reach peak power and percentage decrement of power (*p* < 0.001, *p* < 0.001, *p* = 0.032 and *p* < 0.001, respectively), but not for fatigue index (*p* > 0.05; Figure 3). Peak power was higher in NOR compared to HYP and BFR (*p* < 0.001, dz = 0.65 and dz = 0.87, respectively). Peak power was 913 ± 146 W, 936 ± 139 W, and 991 ± 144 W for BFR, HYP, and NOR, respectively. Mean power was also higher in NOR compared to HYP and BFR (*p* < 0.001, dz = 1.21 and dz = 1.23, respectively). Values for mean power were 641 ± 85 W, 656 ± 89 W, and 715 ± 86 W for BFR, HYP, and NOR, respectively. Time to reach peak power was higher in HYP compared to NOR (*p* = 0.039, dz = 0.33). The average values were 2.39 ± 0.58 s, 2.44 ± 0.90 s, and 2.22 ± 0.56 s for BFR, HYP, and NOR. No significant difference was found in fatigue index between conditions (*p* > 0.05). Values were 50.4% ± 9.8%, 51.5% ± 10.1%, and 49.1% ± 7.5% for BFR, HYP, and NOR, respectively. Percentage decrement of power was lower in NOR compared to BFR and HYP (*p* < 0.001, dz = 0.94 and *p* < 0.001, dz = 0.64, respectively), as well as in BFR compared to HYP (*p* = 0.037; dz = 0.30). Values were −20.2% ± 6.9%, −18.3% ± 6.7% and −14.6% ± 4.7% for BFR, HYP, and NOR, respectively (Figure 3).

A main effect of time was found for all these variables (*p* ≤ 0.010), except the percentage decrement of power (*p* = 0.052). According to *post hoc* analyses, peak power was higher in the first set compared to the second and third sets (*p* < 0.001, dz = 0.98 and *p* < 0.001, dz = 1.23, respectively), as well as in the second set compared to the third (*p* = 0.005, dz = 0.91). Values were 1,013 ± 129 W, 934 ± 137 W and 893 ± 148 W for sets one, two and three, respectively. Mean power was also higher in the first set compared to the second and third sets (*p* < 0.001, dz = 0.91 and *p* < 0.001, dz = 1.31, respectively), as well as in set two compared to set three (*p* < 0.001, dz = 1.07). Values were 714 ± 83 W, 665 ± 86 W and 633 ± 90 W for sets one, two and three, respectively. Time to reach peak power was lower in the third set compared to the first one (*p* = 0.009, dz = 0.51). Values were 2.46 ± 0.74 s, 2.39 ± 0.69 s and 2.21 ± 0.65 s for sets one, two and three, respectively. Finally, fatigue index was higher in the second and third sets compared to the first one (*p* = 0.025, dz = 0.48 and *p* < 0.001, dz = 0.85, respectively). It was also higher in the last set compared to the second set (*p* = 0.024, dz = 0.69). Values were 47% ± 9%, 50% ± 9% and 54% ± 8% for sets one, two and three, respectively. Percentage decrement values were −16.4% ± 6.3%, −18.7% ± 6.0% and −18.0% ± 7.2% for sets one, two and three, respectively.

### Cardiorespiratory responses

During exercise, a main effect of condition was found for ΣVEex, ΣVO_2_ex, ΣVCO_2_ex, and HRex (*p* < 0.001). Post hoc tests revealed that ΣVEex was higher in HYP compared to NOR and BFR (*p* = 0.002, dz = 0.51, and *p* < 0.001, dz = 1.05, respectively). ΣVEex was lower in BFR compared to NOR (*p* < 0.001, dz = 0.71). ΣVO_2_ex was higher in NOR compared to BFR and HYP (*p* < 0.001, dz = 1.04, and dz = 1.12, respectively). ΣVCO_2_ex was higher in NOR compared to BFR and HYP (*p* < 0.001, dz = 0.80, and *p* < 0.001 dz = 0.84, respectively). HRex was lower in BFR compared to HYP and NOR (*p* < 0.001, dz = 0.65, and *p* < 0.001, dz = 0.82, respectively). Furthermore, ΣVEex, ΣVCO_2_ex and HRex presented a main effect of time (*p* < 0.001). ΣVEex was lower during the first set compared to the second and third sets (*p* < 0.001, dz = 0.93 and dz = 0.78, respectively). ΣVCO_2_ex was higher during the first set compared to the second and third sets (*p* < 0.001, dz = 1.56 and dz = 2.06 respectively), as well as in the second set compared to the third (*p* < 0.001, dz = 1.53). HRex was lower in the first set compared to the second and the third sets (*p* = 0.003, dz = 0.48 and *p* < 0.001, dz = 0.74, respectively).

During recovery, a main effect of condition was found for ΣVErest, ΣVO_2_rest, ΣVCO_2_rest and HRrest (*p* < 0.001, *p* = 0.001, *p* = 0.017 and *p* = 0.007, respectively). According to *post hoc* analyses, ΣVErest was higher in HYP compared to BFR (*p* < 0.001, dz = 0.62). ΣVO_2_rest was lower in BFR compared to NOR and HYP (*p* = 0.002, dz = 0.61 and *p* = 0.005, dz = 0.48, respectively). ΣVCO_2_rest was higher in NOR compared to HYP (*p* = 0.019, dz = 0.57). HRrest was lower in BFR compared to NOR and HYP (*p* = 0.012, dz = 0.43 and *p* = 0.022, dz = 0.44, respectively). A main effect of time was observed for ΣVCO_2_rest and HRrest (*p* < 0.001). Indeed, ΣVCO_2_rest presented higher values after the first set than after the second set (*p* < 0.001, dz = 1.20) and HRrest was lower in the first recovery period compared to the second (*p* < 0.001, dz = 1.35, respectively).

A main effect of condition was found for $\dot{V}$ Epeak, $\dot{V}$ O_2_peak, $\dot{V}$ CO_2_peak and HRpeak (*p* < 0.001). As shown by *post hoc* tests, $\dot{V}$ Epeak was lower in BFR compared to HYP and NOR (*p* < 0.001, dz = 0.69 and *p* = 0.016, dz = 0.40, respectively). $\dot{V}$ Epeak was also higher in HYP compared to NOR (*p* = 0.017, dz = 0.39). $\dot{V}$ O_2_peak was lower in HYP compared to NOR and BFR (*p* < 0.001, dz = 1.47, dz = 0.69, respectively), as well as in BFR compared to NOR (*p* < 0.001, dz = 0.73). $\dot{V}$ CO_2_peak was lower in HYP compared to NOR and BFR (*p* < 0.001, dz = 1.07 and dz = 0.46, respectively). It was also higher in NOR compared to BFR (*p* < 0.001, dz = 0.67). HRpeak was lower in BFR compared to NOR and HYP (*p* < 0.001, dz = 0.72, and *p* < 0.001, dz = 0.58, respectively), as well as in HYP compared to NOR (*p* = 0.035, dz = 0.47). Finally, $\dot{V}$ CO_2_peak presented a main effect of time (*p* < 0.001). It was higher during set number one compared to the second and third sets (*p* < 0.001, dz = 1.99 and dz = 2.45, respectively). $\dot{V}$ CO_2_peak was also higher in set two compared to set three (*p* < 0.001, dz = 1.35; Table 1).

**TABLE 1:** Cardiorespiratory variables during the repeated sprint exercise.

Condition	BFR	HYP	NOR	Condition main effect
ΣVEex (L)	270 ± 41^*$^	312 ± 50^*^	296 ± 52	*p* < 0.001
ΣVErest (L)	274 ± 66^$^	309 ± 83	290 ± 72	*p* < 0.001
$\dot{V}$ Epeak (L·min^−1^)	164 ± 21^*$^	177 ± 23^*^	171 ± 25	*p* < 0.001
ΣVO_2_ex (L)	6.81 ± 0.75^*^	6.60 ± 0.64^*^	7.54 ± 0.91	*p* < 0.001
ΣVO_2_rest (L)	5.30 ± 0.80^*$^	5.64 ± 0.71	5.68 ± 0.77	*p* = 0.001
$\dot{V}$ O_2_peak (L·min^−1^)	3.79 ± 0.52^*$^	3.43 ± 0.39^*^	4.06 ± 0.48	*p* < 0.001
ΣVCO_2_ex (L)	6.47 ± 1.04^*^	6.39 ± 1.06^*^	7.12 ± 1.29	*p* < 0.001
ΣVCO_2_rest (L)	6.77 ± 1.44	6.65 ± 1.14^*^	7.14 ± 1.30	*p* = 0.017
$\dot{V}$ CO_2_peak (L·min^−1^)	3.67 ± 0.67^*$^	3.43 ± 0.63^*^	3.94 ± 0.68	*p* < 0.001
HRex (bpm)	151 ± 14^*$^	157 ± 9	160 ± 9	*p* < 0.001
HRrest (bpm)	122 ± 16^*$^	127 ± 14	127 ± 15	*p* = 0.007
HRpeak (bpm)	167 ± 13^*$^	171 ± 8^*^	174 ± 8	*p* < 0.001
Aerobic contribution (%)	18.8 ± 1.4^$^	17.9 ± 2.5^*^	19.0 ± 1.9	*p* < 0.001

Data are shown as mean ± standard deviation. BFR: bilaterally cuffed blood flow restriction of the lower limbs at 45% of resting arterial occlusive pressure, HYP: simulated hypoxia (fraction of inspired oxygen = 13%), NOR: normoxia. ΣVEex, accumulated ventilation during exercise; ΣVErest, accumulated ventilation during recovery; $\dot{V}$ Epeak, peak minute ventilation; ΣVO_2_ex, accumulated oxygen consumption during exercise; ΣVO_2_rest, accumulated oxygen consumption during recovery; $\dot{V}$ O_2_peak, peak oxygen consumption; ΣVCO_2_ex, exhaled carbon dioxide during exercise; ΣVCO_2_rest, exhaled carbon dioxide during recovery, $\dot{V}$ CO_2_peak, peak exhaled carbon dioxide; HRex, heart rate during exercise; HRrest, HR during recovery; HRpeak, peak heart rate; bpm, beats per minute. ^*^, significantly different from NOR (*p* < 0.05); ^$^, Significantly different from HYP (*p* < 0.05).

There were main effects of condition (*p* < 0.001) and time (*p* < 0.001) on aerobic contribution. Aerobic contribution was lower in HYP compared to NOR and BFR (*p* = 0.002, dz = 0.46 and *p* = 0.005, dz = 0.33, respectively). Aerobic contribution was higher in the second and third sets compared to the first set (*p* < 0.001, dz = 1.28 and dz = 1.37, respectively).

### Neuromuscular fatigue

**FIGURE 4:**
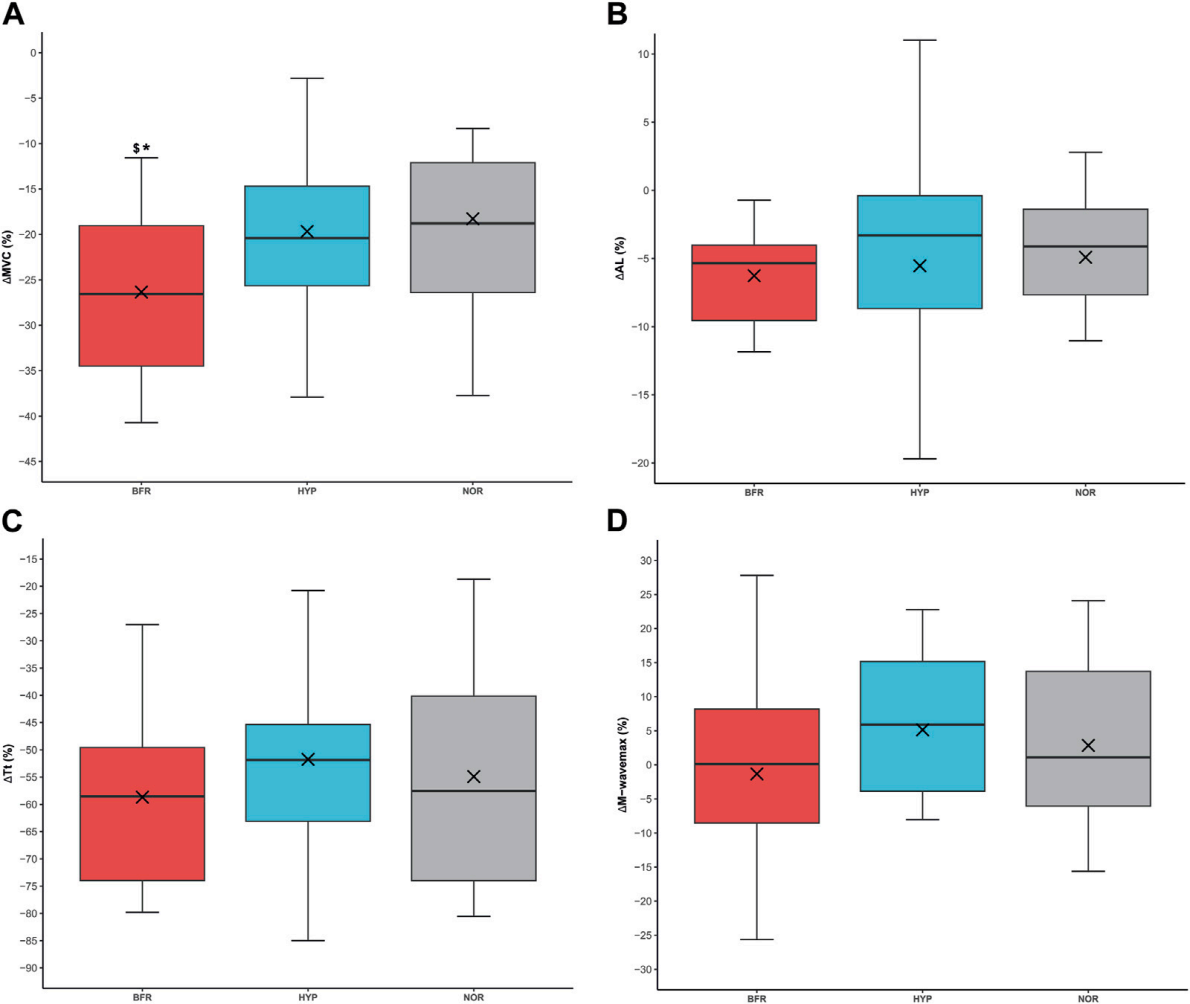
Neuromuscular fatigue under the different conditions. **(A)** Percentage difference in maximal voluntary contraction (ΔMVC). **(B)** Percentage difference in activation level (ΔAL), **(C)** Percentage difference in twitch torque (ΔTt), **(D)** Percentage difference in M-wave_max_ (ΔM-wave_max_). BFR: bilaterally cuffed blood flow restriction of the lower limbs at 45% of resting arterial occlusion, HYP: simulated hypoxia (fraction of inspired oxygen = 13%), NOR: normoxia. ^*^: significantly different from NOR (*p* < 0.05), ^$^: significantly different from HYP (*p* < 0.05). Of note, whisker plots represent the quartiles the horizontal line corresponds to the median and the cross is the mean.

The pre-exercise electrostimulation parameters across all conditions were not significantly different (MVC: 189 ± 30 Nm, 185 ± 30 Nm, and 180 ± 31 Nm (*p* = 0.299); Mwave_max_: 9.7 ± 3.9 mV, 9.9 ± 2.5 mV and 8.6 ± 2.7 mV (*p* = 0.212); AL: 91.6% ± 4.4%, 92.6% ± 3.8%, and 91.0% ± 6.5% (*p* = 0.421); Tt: 64.6 ± 8.2 Nm, 66.5 ± 8.7 Nm, and 60.2 ± 11.7 Nm (*p* = 0.056) for BFR, HYP, and NOR, respectively). A main effect of condition was found for ΔMVC (*p* = 0.008). Post hoc analyses showed that MVC was more impaired in BFR compared to HYP, and NOR (*p* = 0.027, dz = 0.75, and *p* = 0.011, dz = 0.78, respectively). The average ΔMVC was −26.3% ± 9.9%, −19.7% ± 10.4%, and −18.3% ± 12.4% for BFR, HYP, and NOR, respectively. However, no effect was found for ΔAL, ΔTt and ΔMwave_max_ (*p* > 0.05). The average ΔAL was −6.3% ± 6.5%, −5.5% ± 9.0%, and −4.9% ± 8.6% for BFR, HYP, and NOR, respectively. ΔTt values were −58.7% ± 16.6%, −51.8% ± 23.1%, and −54.9% ± 20.4%. Lastly, ΔMwave_max_ was −1.3% ± 15.3%, 5.1% ± 10.6%, and 2.9% ± 13.3% for BFR, HYP, and NOR, respectively (Figure 4).

## Discussion

This crossover trial aimed to compare acute responses of an RSE performed under systemic hypoxia (HYP) or with blood flow restriction (BFR). The main findings were that BFR and HYP impaired power output, BFR was less demanding at the cardiorespiratory level, but MVC was more impaired in BFR compared to the other conditions, evidencing a greater neuromuscular fatigue. Ventilation increased in HYP probably to partly compensate for the decrease in oxygen consumption found in this condition. Moreover, HYP enhanced anaerobic contribution during the current RSE protocol, even though the difference was small (∼1%).

Both BFR and HYP provoked a decrease in power output during the sprint exercise. This was already observed in a study that used the same BFR pressure (i.e., 45% of resting arterial occlusive pressure) (Willis et al., 2018). This latter study used a single-set protocol until exhaustion, and the workload was significantly reduced with BFR due to a significant decrease in the number of sprints. In a study with a fixed number of sprints, power output decreased in hypoxia, which is in agreement with the current study. Furthermore, Billaut et al. (2013) observed an increase in central fatigue (i.e., the activation level decreased further in hypoxia) and similar peripheral fatigue (peak twitch torque was similarly impaired in both conditions). Therefore, the authors suggested that the central nervous system may prevent the development of muscle fatigue, thus impacting power output. Hypoxemia could provoke a decline in the recruitment of motor neurons and/or in their discharge rates. However, no difference in the activation level was found in the current study. On the other hand, when the training volume is not set, studies show that power output can be maintained in hypoxia (Willis et al., 2019a; Willis et al., 2019b; Peyrard et al., 2019). Nevertheless, protocols with longer sprints and recovery periods could be less detrimental to mechanical responses in hypoxia (Kon et al., 2015; Solsona et al., 2021; Takei et al., 2021). Finally, neuromuscular functions were not fully recovered between sets. Indeed, performance variables (i.e., peak power, mean power and fatigue index) were impaired throughout the session excepting time to reach peak power, which was reduced in the third set. This can be explained by the lower peak power observed in the last set, which was then reached faster.

Neuromuscular variables (i.e., ΔAL, ΔTt and ΔMwave_max_) were unchanged within conditions, except ΔMVC, which is a general fatigue index (Huang et al., 2017). MVC decreased further in BFR compared to the other conditions. This could mean that BFR provoked non-significant perturbations in the different stages of fatigue (i.e., corticospinal and sarcolemma excitability, and muscle contractibility), integrating into higher global neuromuscular fatigue. Accordingly, BFR was the condition that induced the greater percentage decrement of power output. Contrastingly, Peyrard and colleagues observed an alteration of peripheral indices (i.e., ΔMwave_max_ and doublet-evoked force) thanks to electrical nerve stimulation after RSE with BFR (Peyrard et al., 2019). However, in their protocol in the upper limbs, Peyrard and coworkers evaluated fatigue 2 minutes after exhaustion, while in the current study, the number of sprints was standardized among conditions and neuromuscular testing took place 1 minute after the last sprint of the session. Furthermore, the main difference between the two protocols is that the authors also used BFR during electrical nerve stimulation, while BFR was not applied during the measurements of neuromuscular fatigue in the present protocol. Nevertheless, ΔAL was not different between NOR, BFR and HYP in both studies. Importantly, the reduced levels of average peak power output found in BFR could be attributable to higher neuromuscular fatigue. However, as no difference was found within conditions (except in ΔMVC between BFR and NOR), it can be hypothesized that the delay between the last sprint and the MVC sufficed to dissipate the difference between conditions. In this sense, in a study it was found that five consecutive 10-s sprints produce peripheral fatigue, and beyond this threshold (i.e., from sprints 6–10), the central mechanisms of fatigue are also involved (Pearcey et al., 2015). Hence, the design of the current study (5 consecutive sprints interspaced by long recoveries, i.e., 5 min) may have prevented the development of important levels of central fatigue, which were then unchanged amongst conditions.

Regarding cardiorespiratory parameters, the current study showed that ΣVEex increased in HYP and decreased in BFR. This result agrees with previous literature showing that BFR at 60% of resting arterial occlusive pressure during RSE reduced ventilation (Willis et al., 2018). However, the latter study found no difference in ventilation at 45% of resting arterial occlusive pressure. It has recently been shown that BFR application provokes the reduction of ventilation and gas exchanges during longer sprint exercises (Solsona et al., 2021). In this study, $\dot{V}$ O_2_peak and HR during exercise were also lower in BFR compared to NOR (Solsona et al., 2021). During the current protocol, $\dot{V}$ Epeak and ΣVO_2_rest was higher in NOR and HYP compared to BFR which means that BFR was less demanding at the respiratory level. The lower cardiorespiratory demands might be related to the lower power output that was observed. Of note, there was a main effect of time (*p* < 0.001) on cardiorespiratory responses (i.e., ΣVEex, ΣVCO_2_ex, ΣVCO_2_rest, $\dot{V}$ CO_2_peak, HRex, HRrest, and aerobic contribution) commonly observed in literature (supplementary data).

Concerning hypoxia, the current study showed that ventilatory responses (i.e., $\dot{V}$ Epeak and ΣVEex) increased in HYP compared to NOR. This suggests a higher load for the ventilatory muscles. However, gas exchanges were reduced or unchanged in HYP compared to NOR. Indeed, $\dot{V}$ O_2_peak and $\dot{V}$ CO_2_peak were lower in HYP compared to NOR. Hence, even though $\dot{V}$ Epeak was higher in HYP, both $\dot{V}$ O_2_peak and $\dot{V}$ CO_2_peak remained lower in this condition. Similarly, the higher ΣVEex observed in HYP did not change ΣVO_2_ex or ΣVCO_2_ex compared to NOR. Studies on RSE showed no difference in ventilation, even though they used slightly lower simulated altitudes (i.e., FiO_2_ = 13.1%) (Willis et al., 2019a; Willis et al., 2019b). Of note, aerobic contribution increases with the repetition of sprints (Bogdanis et al., 1996). Therefore, it can be hypothesized that the higher workload performed in NOR resulted in similar minute ventilation compared to HYP. Finally, the present study demonstrated a reduction of aerobic contribution to energy production with HYP. Accordingly, previous research has shown that acute exposure to hypoxia stimulates muscle sympathetic nerve activity (Xie et al., 2001) and increases circulating catecholamines during exercise (Escourrou et al., 1984). The stimulation of β_2_-adrenergic receptors leads to enhanced glycogenolysis/glycolysis in skeletal muscle (Hostrup et al., 2014; Cairns and Borrani, 2015). Hence, alterations in adrenergic activity could explain enhanced glycolysis in HYP.

### Limitations and future directions

These findings have to be interpreted with caution as first, they only apply to a young, healthy and moderately trained men population and second, a demanding exercise protocol was used. Thus, direct generalization of these results to women or other less trained populations should be approached with caution. Then, there are some limitations to consider when applying BFR. In most studies, resting arterial occlusive pressure is used to individualize the exerted pressure. However, it should be noted that increased blood pressure during exercise may result in a shift in the relative level of restriction (Jessee et al., 2017). Consequently, the applied pressure may not remain constant throughout the session. Regarding post-exercise electrostimulation, the evaluation was performed 1 minute after the last sprint, which may have allowed a partial dissipation of fatigue (particularly central fatigue) and could have minimized the differences between conditions. Nonetheless, this delay is inevitable when a transfer to a different set up is needed (Collins et al., 2018). Despite the absence of a formal familiarization session, participants were physically active and were familiar with high-intensity cycling. Furthermore, the warm-up sprints provided participants with the opportunity to sprint at their individualized torque, serving as a practice before the first trial. As for the electrostimulation tests, the study’s crossover design effectively mitigates this potential bias. Finally, this investigation did not include females because several responses such as cardiovascular adaptations differ with males (Patel et al., 2021) and could introduce variability. Thus, extrapolating from these data presents limitations.

## Conclusion

According to the present study, adding BFR and HYP stresses to our RSE protocol negatively impacted power output. Importantly, BFR provoked higher neuromuscular fatigue and reduced cardiorespiratory constraints. In contrast, HYP did not induce alteration in neuromuscular function but increased the ventilatory response during exercise, as well as peak minute ventilation. Moreover, HYP also increased anaerobic energy contribution during the present RSE protocol compared to normoxia.

## Figures and Tables

**Table:** 

**AL**	activation level
**BFR**	blood flow restriction
**D**	difference between the torque level just before the double twitch and the maximum torque during the double twitch
**EMG**	electromyography
**HRex**	heart rate during exercise
**HRrest**	heart rate during rest
**HYP**	hypoxia
**MVC**	maximal voluntary contraction
**NMES**	neuromuscular electrical stimulation
**NOR**	normoxia
**RSE**	repeated sprint exercise
**RSH**	repeated sprints in hypoxia
**RST**	repeated sprint training
**T**_**b**_	torque before the superimposed doublet
**T**_**DTW**_	torque reached with the isolated doublet
**T**_**max**_	maximal torque during maximal voluntary contraction
$\overset{.}{V}$ **CO**_**2**_ **peak**	peak exhaled carbon dioxide
$\overset{.}{V}$ **Epeak**	peak ventilation
$\overset{.}{V}$ **O**_**2**_ **max**	maximal oxygen uptake
**ΔAL**	percentage difference in activation level
**ΔMVC**	percentage difference in maximal voluntary contraction
**ΔMwave**_**max**_	percentage difference in maximal M-wave
**ΔTt**	percentage difference in twitch torque
**ΣVCO**_**2**_ **ex**	cumulated exhaled carbon dioxide during exercise
**ΣVCO**_**2**_ **rest**	cumulated exhaled carbon dioxide during rest
**ΣVEex**	cumulated ventilation during exercise
**ΣVErest**	cumulated ventilation during rest
**ΣVO**_**2**_ **ex**	cumulated oxygen uptake during exercise
**ΣVO**_**2**_ **rest**	cumulated oxygen uptake during rest

## Data Availability

The raw data supporting the conclusions of this article will be made available by the authors, without undue reservation.
