# Supplementary material for: Impact of systemic hypoxia and blood flow restriction on mechanical, cardiorespiratory, and neuromuscular responses to a multiple-set repeated sprint exercise
Source: Front Physiol. 2024 Jan 31;15:1339284. doi: 10.3389/fphys.2024.1339284 (PMC10864669; doi:10.3389/fphys.2024.1339284)
Supplement: Supplementary file 1 [file Table1.docx]

**Supplementary data**

**Cardiorespiratory variables between the sets**

| **Set** | 1 | 2 | 3 | Set main effect |
| --- | --- | --- | --- | --- |
| ΣVEex (L) | 280±50^2,3^ | 299±51 | 299±50 | p<0.001 |
| ΣVErest (L) | 285±73 | 296±77 | - | p=0.115 |
| V̇Epeak (L·min^-1^) | 171±25 | 169±24 | 172±23 | p=0.527 |
| ΣVO_2_ex (L) | 7.00±0.80 | 7.08±0.85 | 6.89±0.95 | p=0.238 |
| ΣVO_2_rest (L) | 5.49±0.77 | 5.58±0.77 | - | p=0.327 |
| V̇O_2_peak (L·min^-1^) | 3.79±0.52 | 3.76±0.52 | 3.73±0.50 | p=0.582 |
| ΣVCO_2_ex (L) | 7.66±1.11^2,3^ | 6.48±0.76^3^ | 5.85±0.82 | p<0.001 |
| ΣVCO_2_rest (L) | 7.25±1.38^2^ | 6.45±1.10 | - | p<0.001 |
| V̇CO_2_peak (L·min^-1^) | 4.28±0.62^2,3^ | 3.52±0.49^3^ | 3.25±0.49 | p<0.001 |
| HRex (bpm) | 153±11^23^ | 157±12 | 159±11 | p<0.001 |
| HRrest (bpm) | 122±15^2^ | 129±14 | - | p<0.001 |
| HRpeak (bpm) | 169±9 | 170±11 | 172±10 | p=0.122 |
| Aerobic contribution (%) | 17.4±1.7^2,3^ | 18.9±1.9 | 19.3±2.0 | p<0.001 |

*Data are shown as mean±standard deviation. ΣVEex: accumulated ventilation during exercise, ΣVErest: accumulated ventilation during recovery, V̇Epeak: peak minute ventilation, ΣVO_2_ex: accumulated oxygen consumption during exercise, ΣVO_2_rest: accumulated oxygen consumption during recovery, V̇O_2_peak: peak oxygen consumption, ΣVCO_2_ex: exhaled carbon dioxide during exercise, ΣVCO_2_rest: exhaled carbon dioxide during recovery, V̇CO_2_peak: peak exhaled carbon dioxide, HRex: heart rate during exercise, HRrest: HR during recovery, HRpeak: peak heart rate, bpm: beats per minute. ^2^: significantly different from set 2, ^3^: significantly different from set 3*
